# Solvent‐Dependent Biological Activities of Aqueous and Ethanolic Extracts From Edible Insect Larvae

**DOI:** 10.1002/fsn3.71848

**Published:** 2026-05-03

**Authors:** Min‐Cheol Kang, Seung Tae Im, Tae‐Kyung Kim, Min Kyung Park, Hae In Yong, Ginnae Ahn, Eui Jeong Han, Ji‐Yoon Cha, Kyung‐Mo Song, Yun‐Sang Choi

**Affiliations:** ^1^ Research Group of Food Processing Korea Food Research Institute Wanju Republic of Korea; ^2^ Division of Animal and Dairy Sciences Chungnam National University Daejeon Republic of Korea; ^3^ Department of Food Technology and Nutrition Chonnam National University Yeosu Republic of Korea; ^4^ Gwangju Center Korea Basic Science Institute Gwangju Republic of Korea; ^5^ Department of Food Science & Biotechnology Sungshin Women's University Seoul Republic of Korea

**Keywords:** *Allomyrina dichotoma*, edible insect larvae, multifunctional food resource, *Protaetia brevitarsis seulensis*, solvent‐dependent extraction, *Tenebrio molitor*

## Abstract

The increasing demand for sustainable protein sources has intensified interest in edible insects as potential multifunctional food ingredients. In this study, we investigated whether solvent‐dependent extraction influences the biological activities of edible insect larval extracts. Aqueous and ethanolic extracts prepared from the larvae of *Protaetia brevitarsis seulensis* (PBS), *Tenebrio molitor* (TM), and *Allomyrina dichotoma* (AD) were evaluated using multiple in vitro models. The aqueous extracts of AD and PBS exhibited strong antioxidant activity in HepG2 hepatocytes and human dermal fibroblast (HDF) cells. Most extracts suppressed nitric oxide production in lipopolysaccharide‐stimulated RAW 264.7 macrophages, indicating anti‐inflammatory effects. In addition, all larval extracts inhibited β‐hexosaminidase release in IgE/BSA‐stimulated mast cells, suggesting anti‐allergic potential. The protein‐rich aqueous and ethanolic extracts showed relatively stronger angiotensin‐converting enzyme (ACE) inhibitory activity, whereas polyphenol‐rich ethanolic extracts more effectively reduced lipid accumulation in differentiated 3T3‐L1 adipocytes. These findings highlight solvent‐dependent functional activity profiles of edible insect larval extracts and support their potential as sustainable sources of bioactive ingredients.

## Graphical abstract

This study compares the functional activity profiles of aqueous and ethanolic extracts from edible insect larvae. Solvent‐dependent extraction resulted in distinct bioactivity patterns, highlighting the importance of extraction conditions in determining the functional potential of insect‐derived bioactive compounds.

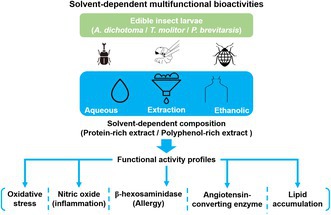

**Abbreviations**
ACE: angiotensin‐converting enzyme
AD: *Allomyrina dichotoma*
BMDMCs: bone marrow‐derived mast cells
HDF: human dermal fibroblast
NO: nitric oxide
PBS: *Protaetia brevitarsis seulensis*
TM:
*Tenebrio molitor*

## 1 Introduction

Global food security remains a critical challenge driven by rapid population growth, climate change, and increasing pressure on natural resources (Saccone and Vallino 2025). According to recent United Nations projections, the global population is expected to approach or exceed nine billion within the next three decades, necessitating a substantial increase in food production to meet future demand (Becker and Fanzo 2023). However, conventional agricultural systems face significant limitations, including reduced arable land availability, high greenhouse gas emissions, excessive water consumption, and unsustainable resource use associated with livestock production (Sumberg and Giller 2022). These constraints underscore the urgent need to develop innovative and sustainable protein sources that can alleviate the environmental burden of traditional animal farming (Food and Agriculture Organization (FAO), et al. 2017). In this context, alternative protein sources such as plant‐based proteins, cultured meat, and edible insects have gained increasing scientific and industrial attention as viable strategies to support global food security (Lisboa et al. 2024; Thornton et al. 2023).

Among alternative protein sources, edible insects have attracted considerable attention due to their high nutritional value, efficient feed conversion, and relatively low environmental footprint compared to conventional livestock. Many edible insect species contain protein levels comparable to or exceeding those of beef, along with substantial amounts of unsaturated fatty acids, vitamins, and essential minerals (Anyasi et al. 2025). Beyond their nutritional composition, edible insects are also recognized as sources of diverse bioactive compounds, including peptides, polyphenols, chitin, and antimicrobial substances. These components have been associated with a range of biological activities, such as antioxidant, anti‐inflammatory, and metabolic regulatory effects, as reported in recent studies (Sanchez‐Estrada et al. 2024). In several East Asian countries, certain edible insect species have been traditionally consumed not only as food but also for their perceived medicinal benefits (Kim, Yong, Kim, et al. 2019; Tang et al. 2019), and recent research has begun to explore these functional properties using in vitro and in vivo models (D'Antonio et al. 2023). Among the diverse edible insect species, *Allomyrina dichotoma* (AD), *Tenebrio molitor* (TM), and *Protaetia brevitarsis seulensis* (PBS) are widely consumed and commercially cultivated in East Asia, making them promising candidates for the development of sustainable functional food ingredients (Kim et al. 2021).

Previous studies have demonstrated that extracts and protein hydrolysates derived from edible insects exhibit diverse biological activities, including antioxidant, antihypertensive, anti‐inflammatory, and metabolic regulatory effects (D'Antonio et al. 2023). Extracts of *A. dichotoma* have been reported to exert protective effects in various experimental models (Pyo et al. 2020; Zielińska et al. 2017), while hydrolysates from *T. molitor* have shown ACE‐inhibitory and immunomodulatory activities associated with specific peptide fractions (Dai et al. 2013). Similarly, bioactive compounds from *P. brevitarsis* have been linked to antioxidant and hepatoprotective effects (Lee, Ryu, et al. 2017; Lee, Kim, Go, et al. 2023). Moreover, a previous study reported that insect‐derived proteins and peptides possess antimicrobial, antioxidant, and antihypertensive activities (De Castro et al. 2018). However, most previous investigations have focused on individual insect species, specific fractions, or single biological endpoints, and relatively few studies have systematically compared solvent‐dependent extraction effects across multiple edible insect larvae under standardized experimental conditions (Di Mattia et al. 2019; Ma et al. 2023). In particular, the influence of solvent polarity on the distribution of protein‐ and polyphenol‐associated bioactivities remains insufficiently characterized.

Therefore, this study aimed to systematically evaluate and compare the functional biological activities of aqueous and ethanolic extracts prepared from the larvae of *A. dichotoma* , *T. molitor* , and *P. brevitarsis seulensis*. A range of in vitro assays was employed to assess antioxidant, antihypertensive, anti‐adipogenic, hepatoprotective, immune‐related, anti‐inflammatory, and anti‐allergic activities under standardized experimental conditions. Through this comparative approach, we sought to examine how solvent‐dependent extraction influences the functional profiles of edible insect larval extracts.

## 2 Materials and Methods

### 2.1 Preparation of Larval Extracts

Freeze‐dried larvae of AD, TM, and PBS were purchased from a commercial insect farm (Farmbang, Sunchang, Korea) and stored at −20°C until use. The samples were ground into fine powder using a laboratory blender. For ethanolic extraction, 3 g of the powdered sample was mixed with 100 mL of 70% ethanol and incubated at room temperature for 24 h with gentle shaking. The extract was filtered through Whatman No. 1 filter paper, and ethanol was removed under reduced pressure using a rotary evaporator. The resulting residue was dissolved in dimethyl sulfoxide (DMSO).

For aqueous extraction, 3 g of larval powder was extracted with 100 mL of hot water (95°C) for 24 h, followed by filtration and freeze‐drying. Hot water extraction at 95°C was employed to maximize the recovery of water‐soluble bioactive compounds and to reduce microbial load (Liceaga 2021). Although high temperatures may induce partial protein denaturation, heat‐stable compounds can retain biological activity (Renard 2004; Jang et al. 2020). The dried extract was reconstituted in DMSO. For all cell‐based assays, the final concentration of DMSO in culture media was maintained below 0.1%.

All extraction procedures were conducted under sterile conditions. Although endotoxin levels were not directly quantified, only minimal nitric oxide production was observed in unstimulated control cells treated with extracts alone, suggesting minimal endotoxin contamination.

### 2.2 Determination of Extraction Yield and Component Analysis

The extraction yield was calculated as the percentage ratio of the dried extract weight to the initial sample weight. Protein concentration was determined using the Pierce BCA protein assay kit (Thermo Fisher Scientific, Waltham, MA, USA). Total polyphenol content was measured using the Folin–Ciocalteu method, and the results were expressed as milligrams of gallic acid equivalents per gram of extract.

### 2.3 Cell Culture

Primary human dermal fibroblast (HDF) cells, human hepatocellular carcinoma (HepG2) cells, and murine macrophage (RAW 264.7) cells were obtained from the Korean Cell Line Bank (Seoul, Korea). Cells were maintained in Dulbecco's modified Eagle medium (DMEM, Gibco, USA) supplemented with 10% heat‐inactivated FBS, 100 U/mL penicillin, 100 μg/mL streptomycin, and 110 mg/L sodium pyruvate at 37°C in a humidified atmosphere of 95% air and 5% CO_2_. Mouse 3T3‐L1 preadipocytes were purchased from American Type Culture Collection (ATCC, Rockville, MD, USA) and cultured in DMEM containing 10% bovine calf serum and 1% penicillin–streptomycin under identical incubation conditions.

### 2.4 Hydrogen Peroxide Scavenging Activity Assay

Hydrogen peroxide (H_2_O_2_) scavenging activity was determined according to a previously reported method (Heo et al. 2006). Briefly, 100 μL of 0.1 M phosphate buffer (pH 5.0) and 20 μL of H_2_O_2_ were added to each well of a 96‐well plate containing the sample extract. After incubation for 5 min at 37°C, 30 μL of 1.25 mM 2,2′‐azino‐bis (3‐ethylbenzothiazoline‐6‐sulfonic acid) (ABTS) and 30 μL of peroxidase (1 U/mL) were added. Following an additional 10 min incubation at 37°C, the absorbance at 405 nm was measured using a microplate reader (Amersham Pharmacia Biotech, UK). Wells without H_2_O_2_ served as blank controls, while wells containing H_2_O_2_ without extracts were used as negative controls.

### 2.5 Protective Effects Against H_2_O_2_ ‐Induced Cytotoxicity

The concentration ranges used in the present study were determined based on preliminary cytotoxicity screening and previously reported concentration ranges for insect‐derived extracts in similar in vitro models (D'Antonio et al. 2023). The cytoprotective effects of larval extracts were evaluated using an EZ‐Cytox Cell Viability Assay Kit (DoGen, Seoul, Korea). HDF and HepG2 cells were seeded into 96‐well plates (1.0 × 10^5^ cells/well) and pretreated with the extracts (100–400 μg/mL) for 16 h, followed by exposure to 1 mM H_2_O_2_ for 24 h. Cell viability was measured by adding EZ‐Cytox solution to each well (final volume 220 μL) and incubating for 3 h at 37°C. Absorbance was read at 450 nm using a microplate reader.

### 2.6 Anti‐Inflammatory Activity in RAW 264.7 Cells

RAW 264.7 cells were seeded at 1.0 × 10^5^ cells/well in 96‐well plates and incubated for 24 h prior to treatment with 100, 200, and 400 μg/mL of each larval extract. Nitric oxide (NO) production was determined by Griess assay. Equal volumes (100 μL) of culture supernatant and Griess reagent (1% sulfanilamide and 0.1% naphthylethylenediamine dihydrochloride in 2.5% phosphoric acid) were mixed and incubated for 10 min at room temperature. Absorbance was recorded at 540 nm, and untreated cells served as the control.

### 2.7 Cytotoxicity and β‐Hexosaminidase Release in Bone Marrow‐Derived Mast Cells (BMDMCs)

BMDMCs were obtained from C57BL/6 mouse bone marrow cells following a previously described method (Han et al. 2020). Bone marrow cells were cultured in DMEM containing 10% FBS, 1% penicillin–streptomycin, 10% pokeweed mitogen‐stimulated spleen cell‐conditioned medium (PWM‐SCM), and 0.2% 2‐mercaptoethanol (Sigma‐Aldrich, St, Louis, Mo, USA) at 37°C in 5% CO_2_. The medium was replaced weekly for 8 weeks until > 98% of the cells were confirmed as mast cells by Giemsa staining. For cytotoxicity testing, cells (2 × 10^4^/well) were seeded in 96‐well plates and treated with larval extracts (15.6–62.5 μg/mL) for 24 h. Lower concentration ranges were applied in mast cell assays due to higher cellular sensitivity. Following incubation, 15 μL of MTT solution (5 mg/mL) was added and incubated for 4 h. Formazan crystals were dissolved in 150 μL of DMSO, and absorbance was measured at 540 nm (Tecan Sunrise, Grödig, Austria). β‐hexosaminidase release was assessed in BMDMCs sensitized with anti‐DNP‐IgE and stimulated with DNP‐BSA in the presence or absence of the extracts, as previously described (Han et al. 2020).

### 2.8 Angiotensin‐Converting Enzyme (ACE) Inhibitory Activity

ACE‐inhibitory activity was measured using a colorimetric ACE Kit‐WST (Dojindo, Japan) following the manufacturer's protocol (Kim et al. 2020). The assay quantifies 3‐hydroxybutyric acid formed from 3‐hydroxybutyryl‐Gly‐Gly‐Gly substrate hydrolysis by ACE, and results were expressed as a percentage of inhibition relative to the control. ACE inhibitory activity was calculated according to the manufacturer's instructions using the provided formula.

### 2.9 Inhibition of Adipogenesis of 3T3‐L1 Adipocytes

3T3‐L1 preadipocytes were seeded in 6‐well plates (4 × 10^5^ cells/well) into and cultured until confluence. Differentiation was induced using DMEM supplemented with 10% FBS, 0.5 mM isobutylmethlxanthine (IBMX), 0.25 μM dexamethasone (Dex), and 10 μg/mL insulin, with or without the larval extracts. After 2 days, the medium was replaced with DMEM containing 10% FBS and 5 μg/mL insulin, refreshed every 2 days. On Day 8, lipid accumulation was visualized by Oil Red O staining. Cells were fixed with 10% formalin, stained with 0.5% Oil Red O in isopropanol for 60 min, and washed with PBS. The retained dye was extracted with isopropanol and quantified by measuring absorbance at 520 nm.

### 2.10 Statistical Analysis

All experiments were conducted with at least three independent biological replicates, and each treatment condition was measured in triplicate wells (technical replicates). Data were expressed as means ± standard deviation (SD). Statistical analyses were conducted using SPSS software (version 8.0; IBM Corp., Armonk, NY, USA). One‐way analysis of variance (ANOVA) followed by Duncan's multiple range test was used to determine statistical significance. Duncan's multiple range test was selected for post hoc comparisons due to its suitability for detecting differences among multiple treatment groups in exploratory biological studies. A *p* < 0.05 was considered statistically significant.

## 3 Results and Discussion

### 3.1 Extraction Yield and Component Analysis of Larval Extracts

The extraction yield, protein, and polyphenol contents of the aqueous and ethanolic larval extracts are summarized in Table 1. Among the aqueous extracts, PBS exhibited the highest yield, while its ethanolic extract also showed the greatest extraction efficiency (24.44% ± 3.85%). Differences in extraction yield may reflect species‐specific compositional characteristics as well as solvent polarity‐dependent solubility of extractable constituents (Rahman et al. 2024; Tourabi et al. 2025).

**TABLE 1:** Extraction yield and compositional characteristics (protein and total polyphenol contents) of edible insect larval extracts.

Sample	*Allomyrina dichotoma* (AD)		*Tenebrio molitor* (TM)		*Protaetia brevitarsis seulensis* (PBS)	
Solvent	DW	70% E	DW	70% E	DW	70% E
Yield (%)	37.78 ± 1.92	22.22 ± 1.92	37.78 ± 1.92	16.67 ± 0.00	50.00 ± 0.00	24.44 ± 3.85
Protein content (mg/mL)	0.68 ± 0.01	0.70 ± 0.01	0.57 ± 0.01	0.40 ± 0.00	0.58 ± 0.02	0.56 ± 0.01
Polyphenol content (μg/mL)	39.94 ± 0.82	53.82 ± 0.74	30.81 ± 0.35	31.96 ± 1.14	33.84 ± 0.96	51.72 ± 2.89

*Note:* DW: aqueous; 70% E: ethanolic.

The protein contents of the ethanolic extracts of AD, TM, and PBS were 0.70 ± 0.01, 0.40 ± 0.00, and 0.56 ± 0.01 mg/mL, respectively, whereas the aqueous extracts contained 0.68 ± 0.01, 0.57 ± 0.01, and 0.58 ± 0.02 mg/mL. The relatively higher protein concentration observed in the aqueous extract of AD is consistent with previous reports indicating that AD larvae contain abundant water‐soluble protein fractions (Kim, Yong, Jeong, et al. 2019).

As expected, ethanolic extraction generally resulted in higher total polyphenol concentrations compared to aqueous extraction. Ethanol, owing to its intermediate polarity, has been widely reported to enhance the recovery of phenolic and moderately lipophilic compounds from natural matrices (Altemimi et al. 2017; Khoddami et al. 2013). Notably, the ethanolic extracts of AD and PBS contained greater polyphenol levels than their aqueous counterparts, indicating that solvent selection influences the compositional profiles of the extracts.

Although only total protein and total polyphenol contents were quantified in the present study, these measurements provide a general characterization of the extracts. The observed compositional differences may serve as reference information for interpreting variations in biological activities across different extracts, although direct attribution to specific compounds requires further investigation.

### 3.2 Anti‐Oxidant Effects of Larval Extracts

Reactive oxygen species (ROS), including superoxide anions (O_2_^−^), hydrogen peroxide (H_2_O_2_), and hydroxyl radicals (•OH), are continuously generated during normal cellular metabolism and play essential roles in redox signaling and host defense (Krumova and Cosa 2016). However, excessive ROS production disrupts cellular redox homeostasis and contributes to oxidative damage to lipids, proteins, and nucleic acids (Jomova et al. 2023; Zhang et al. 2022).

**FIGURE 1:**
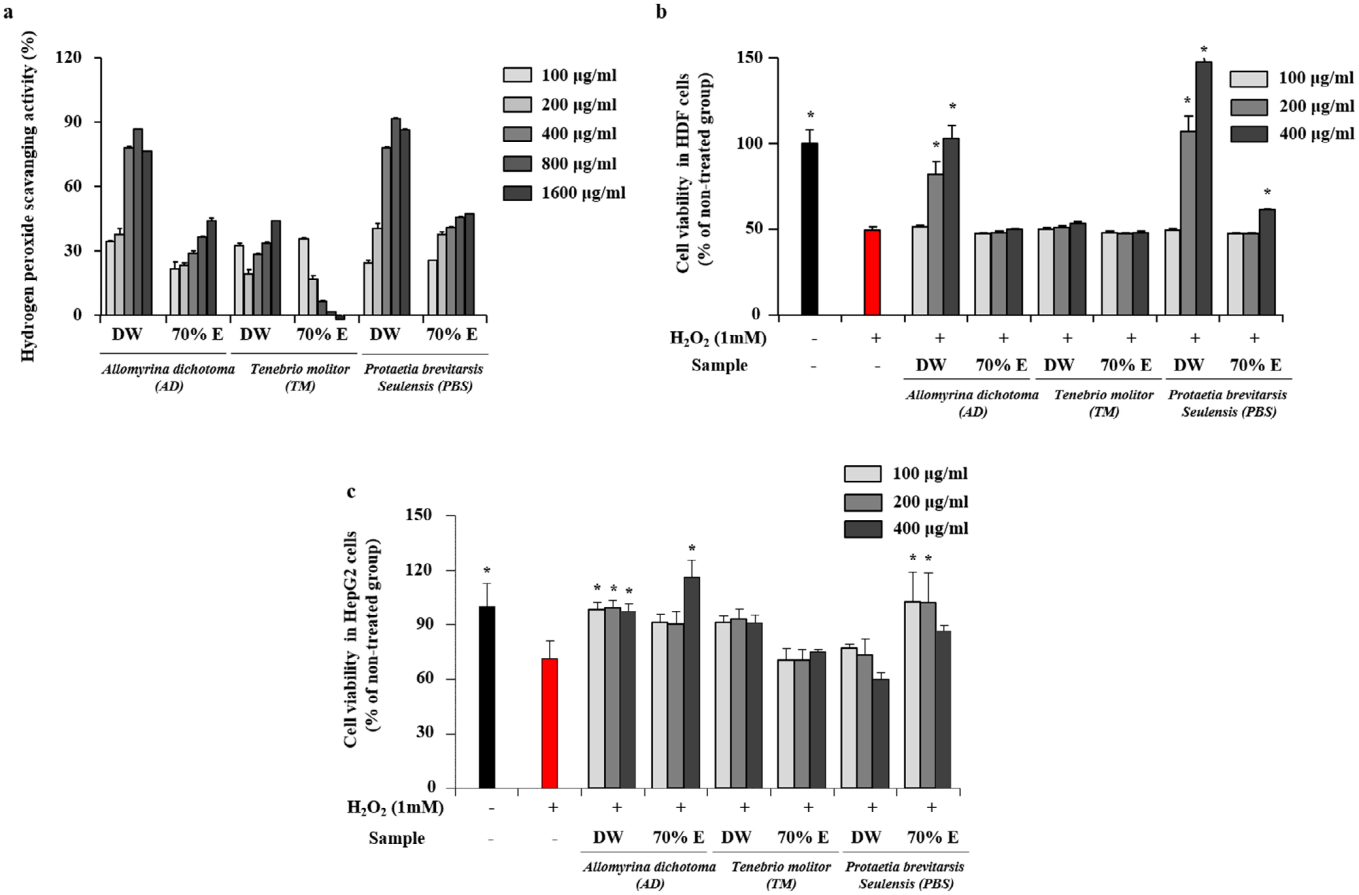
Antioxidant effects of aqueous and ethanolic larval extracts. (a) Hydrogen peroxide (H_2_O_2_) scavenging activity of aqueous and ethanolic extracts. (b) Protective effects of larval extracts (100–400 μg/mL) against H_2_O_2_‐induced oxidative stress in HDF cells. (c) Protective effects of the extracts against H_2_O_2_‐induced cytotoxicity in HepG2 cells under the same treatment conditions. In panels (b) and (c), (−) indicates untreated control cells and (+) indicates cells treated with H_2_O_2_ alone. Data are expressed as mean ± SD from three independent biological experiments (*n* = 3), each performed in triplicate. Significant differences were determined at *p < 0.05 versus H_2_O_2_‐treated group.

As shown in Figure 1a, hydrogen peroxide scavenging activities differed depending on both insect species and extraction solvent. While solvent type did not significantly affect the scavenging activity of TM extracts, the aqueous extracts of AD and PBS exhibited markedly stronger H_2_O_2_‐neutralizing capacity than their ethanolic counterparts. This result suggests that water extraction may preferentially recover hydrophilic constituents associated with antioxidant activity (Munoz‐Seijas et al. 2025).

To further evaluate cytoprotective effects under oxidative conditions, HDF cells were exposed to H_2_O_2_‐induced oxidative stress (Bai et al. 2021). Treatment with 1 mM H_2_O_2_ reduced cell viability by approximately 50%, confirming effective oxidative injury. Notably, aqueous extracts of AD and PBS significantly improved HDF cell viability in a dose‐dependent manner (Figure 1b). Similar protective effects were observed in H_2_O_2_‐treated HepG2 hepatocytes (Figure 1c), where aqueous extract of AD and ethanolic extract of PBS enhanced cell survival compared to the H_2_O_2_‐treated group.

These findings suggest that solvent‐dependent differences in extract composition may influence antioxidant efficacy. In particular, aqueous extracts of AD and PBS exhibited strong hydrogen peroxide scavenging activity and cytoprotective effects both in HDF and HepG2 cells, indicating that hydrophilic components may play a key role in antioxidant responses. These observations are consistent with previous reports demonstrating that water extracts of *T. molitor* and *P. brevitarsis* exhibit significant radical scavenging activity and cytoprotective effects (Lee et al. 2025; Park and Lee 2023), supporting the contribution of water‐soluble antioxidant constituents in these species.

### 3.3 Immune‐Enhancing Activity and Anti‐Inflammatory Effects of Larval Extracts

**FIGURE 2:**
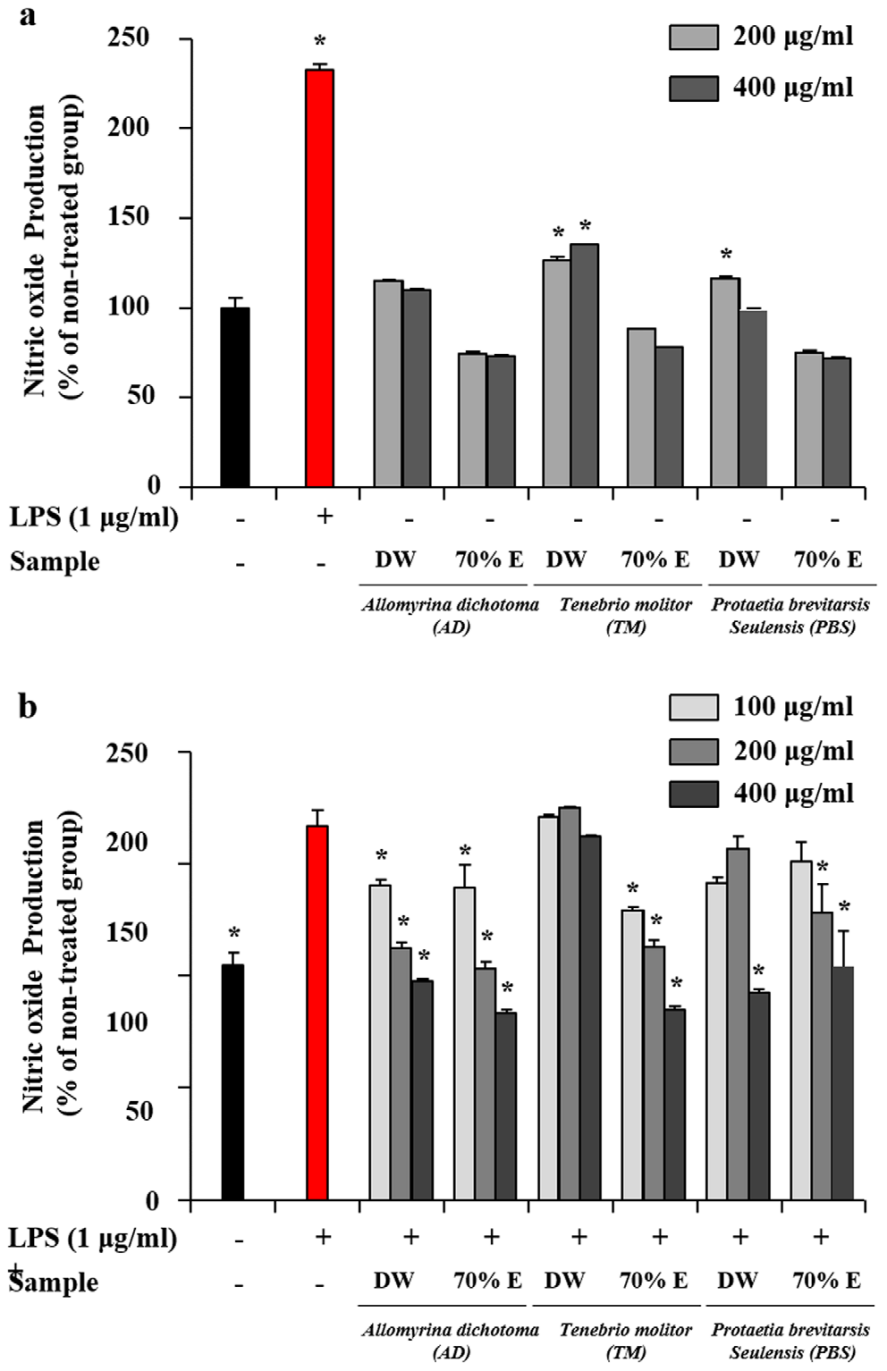
Immune‐enhancing effects and anti‐inflammatory effects of aqueous and ethanolic larval extracts on RAW 264.7 macrophages. (a) Nitric oxide (NO) production in RAW 264.7 cells treated with larval extracts (100–400 μg/mL) for 24 h under unstimulated conditions. (b) Inhibitory effects of the extracts on NO production in lipopolysaccharide (LPS)‐stimulated RAW 264.7 cells. Cells were pretreated with extracts (100–400 μg/mL) for 1 h and subsequently stimulated with LPS (1 μg/mL) for 24 h. In panel (a), untreated cells (−) served as the control group. In panel (b), LPS‐treated cells without extract (+) were used as the inflammatory control. Data are expressed as mean ± SD from three independent biological experiments (*n* = 3), each performed in triplicate. *p < 0.05 versus untreated control (a); *p < 0.05 versus LPS‐treated group (b).

The immune‐enhancing and anti‐inflammatory activities of the larval extracts were evaluated using RAW 264.7 macrophages (Figure 2). Macrophages play a central role in innate immunity by responding to external stimuli and regulating inflammatory responses (Geum et al. 2020). Nitric oxide (NO) production is commonly used as an indicator of macrophage activation and inflammatory mediator release. Under basal conditions, moderate NO generation may reflect immune stimulation, whereas excessive NO production in response to lipopolysaccharide (LPS) is associated with inflammatory responses (Soares et al. 2023).

As shown in Figure 2a, aqueous extracts of TM and PBS significantly increased NO production in unstimulated macrophages, suggesting mild immune activation. Previous studies have reported that components derived from edible insects, including chitin derivatives and protein hydrolysates, can modulate innate immune responses (Rivero‐Pino et al. 2024).

In contrast, inhibition of LPS‐induced NO production is widely used as a marker of anti‐inflammatory activity (Joo et al. 2014). As shown in Figure 2b, both aqueous and ethanolic extracts of AD significantly reduced NO production in LPS‐stimulated macrophages in a concentration‐dependent manner. The ethanolic extract of TM also exhibited a significant inhibitory effect, whereas its aqueous extract showed limited activity. Similarly, both ethanolic and aqueous extracts of PBS markedly suppressed NO production at 400 μg/mL.

These results indicate that the immunomodulatory responses of the extracts vary depending on both extraction solvent and insect species. Notably, aqueous extracts tended to exhibit greater immune‐stimulating effects under basal conditions, whereas ethanolic extracts showed stronger inhibitory effects under inflammatory conditions. The stronger anti‐inflammatory effects observed in ethanolic extracts may reflect the extraction of moderately polar bioactive compounds. Supporting this interpretation, previous studies have identified anti‐inflammatory molecules in *A. dichotoma* larvae, including tetrahydroquinoline derivatives that suppress NF‐κB signaling pathways (Park et al. 2020). These findings indicate that solvent‐dependent extraction influences the distribution of compounds involved in inflammatory regulation.

Overall, the larval extracts demonstrated context‐dependent immunomodulatory properties, characterized by mild activation in unstimulated cells and suppression of excessive NO production in LPS‐stimulated conditions. Further studies are required to identify the specific bioactive components responsible for these effects.

### 3.4 Anti‐Allergic Effects of Larval Extracts on Bone Marrow‐Derived Mast Cells (BMDMCs) Through Inhibition of β‐Hexosaminidase Release

**FIGURE 3:**
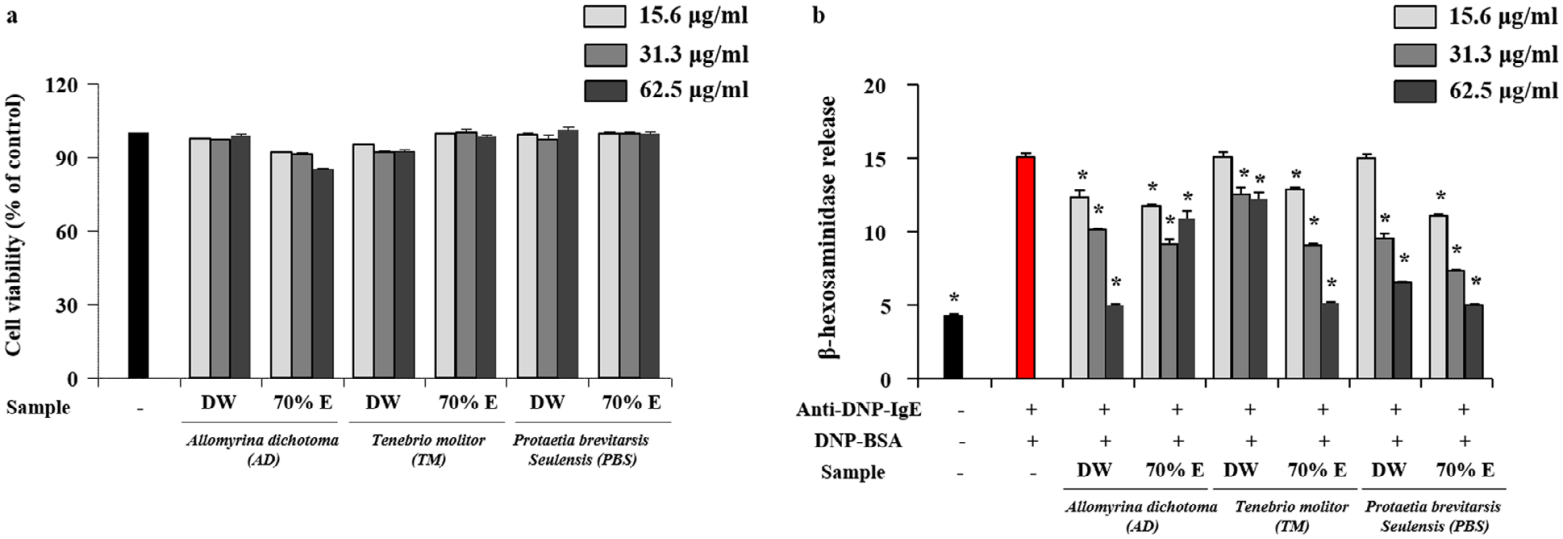
Anti‐allergic effects of aqueous and ethanolic larval extracts on bone marrow‐derived mast cells (BMDMCs). (a) Cytotoxicity of larval extracts (15.6–62.5 μg/mL) in BMDMCs after 24 h treatment, assessed by MTT assay. (b) Inhibitory effects of larval extracts on β‐hexosaminidase release in anti‐DNP‐IgE‐sensitized and DNP‐BSA‐stimulated BMDMCs. Cells were sensitized with anti‐DNP‐IgE (1 μg/mL) for 4 h and subsequently stimulated with DNP‐BSA in the presence or absence of larval extracts. IgE/BSA‐treated cells without extract (+) served as the allergic control group. Data are expressed as mean ± SD from three independent biological experiments (*n* = 3), each performed in triplicate. *p < 0.05 versus IgE/BSA‐treated control.

The anti‐allergic activity of the larval extracts was evaluated in BMDMCs by assessing cytotoxicity and β‐hexosaminidase release (Figure 3). As shown in Figure 3a, treatment with larval extracts at concentrations of 15.6–62.5 μg/mL did not induce cytotoxic effects, confirming that the observed changes in mediator release were not attributable to reduced cell viability.

β‐hexosaminidase is co‐released with histamine during mast cell degranulation and is widely used as a marker of allergic activation (Huanjin et al. 2020). Crosslinking of FcεRI‐bound IgE by antigen (IgE/BSA) triggers intracellular calcium mobilization and the subsequent release of inflammatory mediators. In the present study, IgE/BSA stimulation significantly increased β‐hexosaminidase secretion compared with untreated controls, confirming successful induction of mast cell activation. Co‐treatment with larval extracts markedly suppressed β‐hexosaminidase release (Figure 3b), indicating inhibition of mast cell degranulation.

Although the specific active compounds were not identified in this study, previous reports have suggested that insect‐derived peptides, unsaturated fatty acids, and phenolic compounds may influence mast cell responses (Choi et al. 2024; Lee, Seo, et al. 2017). Notably, the inhibitory effects on β‐hexosaminidase release varied depending on both insect species and extraction solvent. The aqueous extract of AD exhibited a pronounced inhibitory effect, whereas TM showed greater activity in its ethanolic extract. In the case of PBS, both aqueous and ethanolic extracts were effective, with the ethanolic extract demonstrating slightly stronger inhibition.

These observations indicate that anti‐allergic activity is not uniformly distributed across extracts but instead follows a species‐ and solvent‐dependent activity pattern.

### 3.5 Anti‐Hypertensive and Anti‐Obesity Effects of Larval Extracts

Angiotensin‐converting enzyme (ACE) is a key regulator of the renin‐angiotensin‐aldosterone system (RAAS), which modulates vascular resistance and fluid balance. ACE catalyzes the conversion of angiotensin I to angiotensin II, a potent vasoconstrictor implicated in hypertension and cardiovascular dysfunction (Atlas 2007; Tikellis and Thomas 2012). Inhibition of ACE activity is therefore a well‐established strategy for blood pressure control. Numerous studies have reported that bioactive constituents derived from food proteins exhibit ACE‐inhibitory properties (Ko et al. 2017; Xiang et al. 2023). Edible insects are likewise rich in peptides and protein hydrolysates that may contribute to ACE inhibition and cardiovascular protection (Zielińska et al. 2020).

**FIGURE 4:**
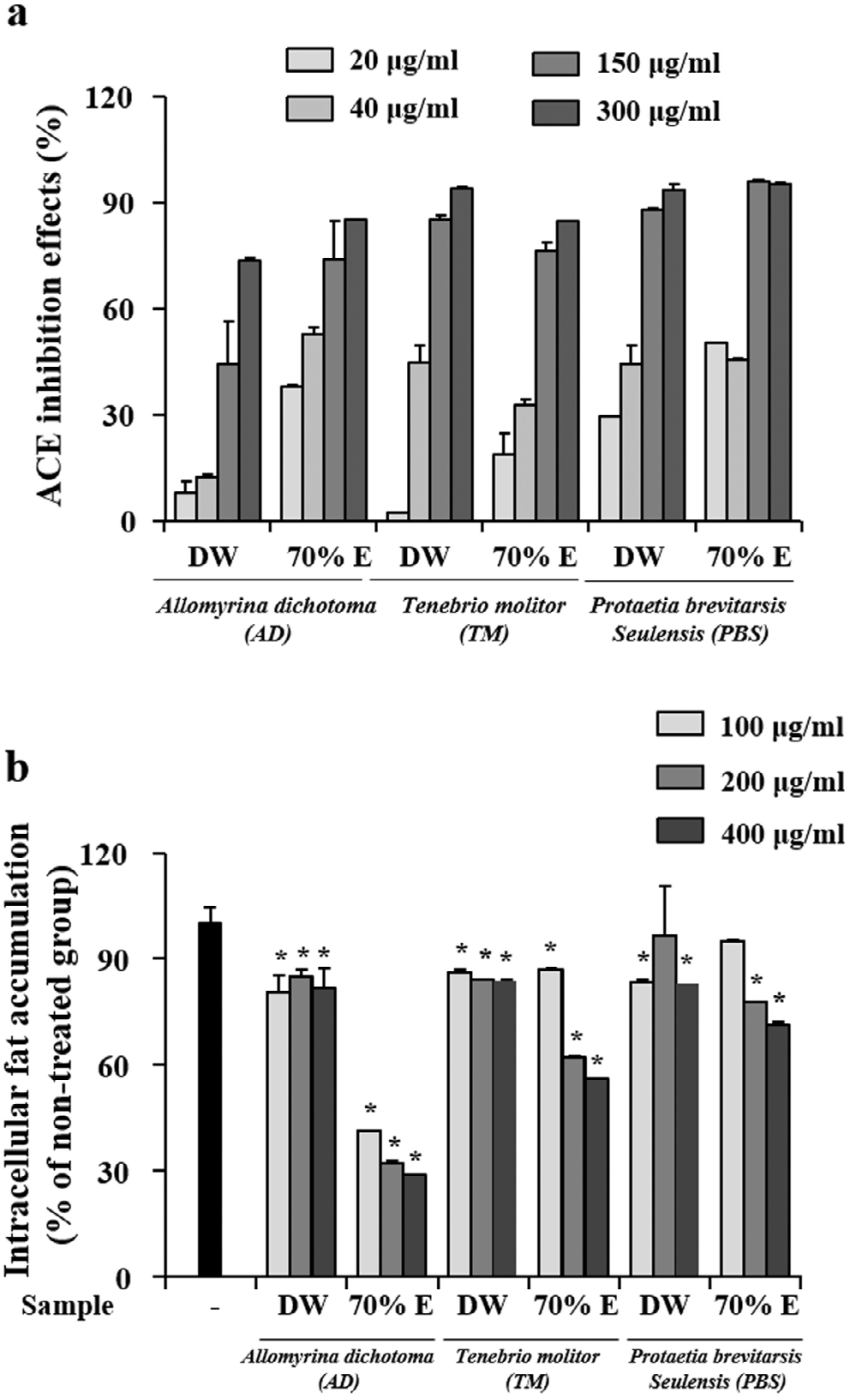
Anti‐hypertensive and anti‐adipogenic effects of aqueous and ethanolic larval extracts. (a) Angiotensin‐converting enzyme (ACE) inhibitory activity of larval extracts at the indicated concentrations (20–300 μg/mL), measured using a colorimetric ACE Kit‐WST assay according to the manufacturer's instructions. Results are expressed as percentage inhibition relative to untreated control. (b) Effects of larval extracts on intracellular lipid accumulation in differentiated 3T3‐L1 adipocytes. Cells were induced to differentiate for 8 days in the presence or absence of extracts (100–400 μg/mL), and lipid accumulation was quantified by Oil Red O staining at 520 nm. Untreated differentiated cells (−) served as control group. Data are expressed as mean ± SD from three independent biological experiments (*n* = 3), each performed in triplicate. *p < 0.05 versus control.

As shown in Figure 4a, both aqueous and ethanolic larval extracts demonstrated ACE‐inhibitory activity. The ethanolic extracts generally exhibited stronger inhibition than the aqueous extracts, with the ethanolic extract of PBS showing the highest activity in a dose‐dependent manner. Notably, the observed ACE‐inhibitory patterns were generally consistent with the distribution of protein content among the extracts. For example, TM extracts exhibited higher protein content in the aqueous fraction and showed stronger ACE inhibition under the same condition, while PBS showed comparable protein levels and similar inhibitory trends across both solvents. These observations suggest a possible association between protein‐rich fractions and ACE‐inhibitory activity, although specific bioactive peptides were not identified in this study. This interpretation is supported by previous studies reporting that protein hydrolysates from *P. brevitarsis* exhibit in vitro ACE inhibitory activity (Lee, Kim, Yong, et al. 2023).

Obesity is a major contributor to metabolic disorders, including hypertension, insulin resistance, and cardiovascular disease (World Health Organization (WHO), et al. 2020). A variety of bioactive insect‐derived compounds have been shown to modulate adipogenesis and lipid metabolism (Lange and Nakamura 2023; Peng et al. 2025; Seo et al. 2017). In the present study, lipid accumulation in differentiated 3T3‐L1 adipocytes was significantly reduced following treatment with larval extracts (Figure 4b). The ethanolic extract of AD exhibited the strongest inhibitory effect on lipid accumulation. In vitro studies have also demonstrated that *A. dichotoma* larvae can alleviate pre‐adipocyte differentiation and lipid accumulation (Chung et al. 2014). Notably, this trend was consistent with the higher polyphenol content observed in ethanolic extracts across all insect species. These results suggest that polyphenol‐enriched fractions may be associated with the observed anti‐adipogenic effects (He et al. 2024), although further chemical characterization is required to confirm this relationship.

Taken together, these findings indicate that solvent‐dependent extraction influences not only the compositional profiles but also the functional activity patterns of edible insect larval extracts. The observed associations between protein content and ACE inhibition, as well as between polyphenol content and anti‐adipogenic activity, highlight the importance of extraction conditions in shaping the biological responses of complex natural extracts. Rather than reflecting a single underlying mechanism, the results indicate assay‐specific and solvent‐dependent activity patterns.

## 4 Conclusions

This study systematically evaluated the functional activities of aqueous and ethanolic extracts derived from the larvae of *Allomyrina dichotoma*, *Tenebrio molitor* , and *Protaetia brevitarsis seulensis* using multiple in vitro models. The results demonstrate that solvent‐dependent extraction significantly influences the biological activity profiles of the larval extracts, with distinct patterns observed across antioxidant, ACE‐inhibitory, anti‐adipogenic, immunomodulatory, anti‐inflammatory, and anti‐allergic assays.

Notably, the distribution of biological activities varied depending on both insect species and extraction solvent. Multiple aqueous and ethanolic extracts generally exhibited strong ACE‐inhibitory activity, whereas ethanolic extracts showed greater anti‐adipogenic effects. These activity patterns were consistent with differences in extract composition, where protein‐rich fractions were associated with ACE inhibition and polyphenol‐enriched fractions were associated with lipid accumulation suppression. Although specific bioactive compounds were not identified, these findings suggest that solvent‐dependent compositional variation may contribute to the observed functional diversity.

Overall, this study highlights the importance of extraction conditions in shaping the functional activity profiles of edible insect larval extracts and supports their potential as sustainable sources of bioactive ingredients. However, further studies involving compound‐level characterization, mechanistic validation, and in vivo evaluation are required to fully elucidate their biological relevance and application potential.

## Author Contributions

**Min‐Cheol Kang:** methodology, writing – original draft, visualization, conceptualization. **Kyung‐Mo Song:** methodology, formal analysis. **Min Kyung Park:** methodology, investigation. **Hae In Yong:** methodology, formal analysis. **Seung Tae Im:** data curation, writing – review and editing. **Tae‐Kyung Kim:** methodology, investigation. **Yun‐Sang Choi:** project administration, supervision, conceptualization. **Ji‐Yoon Cha:** methodology, formal analysis. **Eui Jeong Han:** methodology, investigation. **Ginnae Ahn:** methodology, formal analysis.

## Funding

This work was funded by the Rural Development Administration (Grant RS‐2026‐25508714) and the Ministry of SMEs and Startups (Grant S3452772).

## Ethics Statement

The authors have nothing to report.

## Conflicts of Interest

The authors declare no conflicts of interest.

## Data Availability

The data that support the findings of this study are available from the corresponding author upon reasonable request.
